# Improving Patient Outcomes Following Total Knee Arthroplasty: Identifying Rehabilitation Pathways Based on Modifiable Psychological Risk and Resilience Factors

**DOI:** 10.3389/fpsyg.2020.01061

**Published:** 2020-05-29

**Authors:** Elizabeth Ditton, Sarah Johnson, Nicolette Hodyl, Traci Flynn, Michael Pollack, Karen Ribbons, Frederick Rohan Walker, Michael Nilsson

**Affiliations:** ^1^Centre for Rehab Innovations, The University of Newcastle, Callaghan, NSW, Australia; ^2^Hunter Medical Research Institute, New Lambton Heights, NSW, Australia; ^3^School of Medicine and Public Health, The University of Newcastle, Callaghan, NSW, Australia; ^4^School of Electrical Engineering and Computing, The University of Newcastle, Callaghan, NSW, Australia; ^5^School of Humanities and Social Science, The University of Newcastle, Callaghan, NSW, Australia; ^6^John Hunter Hospital, Hunter New England Local Health District, New Lambton, NSW, Australia; ^7^School of Biomedical Sciences and Pharmacy, Priority Research Centre for Stroke and Brain Injury, The University of Newcastle, Callaghan, NSW, Australia; ^8^NHMRC Centre for Research Excellence in Stroke Rehabilitation and Brain Recovery, Heidelberg, VIC, Australia; ^9^Lee Kong Chian School of Medicine, Nanyang Technological University, Singapore, Singapore

**Keywords:** knee, arthroplasty, recovery, rehabilitation, depression, anxiety, resilience, precision

## Abstract

Total knee arthroplasty (TKA) is a commonly implemented elective surgical treatment for end-stage osteoarthritis of the knee, demonstrating high success rates when assessed by objective medical outcomes. However, a considerable proportion of TKA patients report significant dissatisfaction postoperatively, related to enduring pain, functional limitations, and diminished quality of life. In this conceptual analysis, we highlight the importance of assessing patient-centered outcomes routinely in clinical practice, as these measures provide important information regarding whether surgery and postoperative rehabilitation interventions have effectively remediated patients’ real-world “quality of life” experiences. We propose a novel precision medicine approach to improving patient-centered TKA outcomes through the development of a multivariate machine-learning model. The primary aim of this model is to predict individual postoperative recovery trajectories. Uniquely, this model will be developed using an interdisciplinary methodology involving non-linear analysis of the unique contributions of a range of preoperative risk and resilience factors to patient-centered TKA outcomes. Of particular importance to the model’s predictive power is the inclusion of a comprehensive assessment of modifiable psychological risk and resilience factors that have demonstrated relationships with TKA and other conditions in some studies. Despite the potential for patient psychological factors to limit recovery, they are typically not routinely assessed preoperatively in this patient group, and thus can be overlooked in rehabilitative referral and intervention decision-making. This represents a research-to-practice gap that may contribute to adverse patient-centered outcomes. Incorporating psychological risk and resilience factors into a multivariate prediction model could improve the detection of patients at risk of sub-optimal outcomes following TKA. This could provide surgeons and rehabilitation providers with a simplified tool to inform postoperative referral and intervention decision-making related to a range of interdisciplinary domains outside their usual purview. The proposed approach could facilitate the development and provision of more targeted rehabilitative interventions on the basis of identified individual needs. The roles of several modifiable psychological risk and resilience factors in recovery are summarized, and intervention options are briefly presented. While focusing on rehabilitation following TKA, we advocate for the broader utilization of multivariate prediction models to inform individually tailored interventions targeting a range of health conditions.

## Introduction

Osteoarthritis is a common chronic musculoskeletal condition affecting approximately 9% of Australians (Australian Bureau of Statistics [ABS], 2019) and an estimated 250 million people globally (Vos et al., 2012). Due to the confluence of the increasing prevalence of risk factors such as obesity, aging, and joint injuries, the incidence and prevalence of osteoarthritis are also rising (Hunter and Bierma-Zeinstra, 2019). Symptoms may include severe pain, stiffness, and instability in the affected joint/s (Stark and Price, 2019; Törmälehto et al., 2019). Because there is no known cure and symptoms tend to worsen in severity over time, osteoarthritis can have a progressively debilitating impact on an individual’s health and functioning (Hunter and Bierma-Zeinstra, 2019; Törmälehto et al., 2019), particularly when conservative management interventions are unsuccessful in restricting disease progression (Jones et al., 2007; O’Brien et al., 2019). Patients with end-stage osteoarthritis experience considerable pain, as well as functional limitations in relation to mobility, activities of daily living, independence, and occupational and social participation (Neogi, 2013; O’Brien et al., 2019; Törmälehto et al., 2019). Symptoms can lead to disrupted sleep and fatigue (Sasaki et al., 2014a), and reliance on a caregiver (Hunter et al., 2014). These difficulties affect mood, psychological wellbeing and health-related quality of life (Jones et al., 2007; Sasaki et al., 2014a; Törmälehto et al., 2019).

Osteoarthritis is a major contributor to the global health burden (Hunter and Bierma-Zeinstra, 2019) and one of the highest causes of global disability (Neogi, 2013; Cross et al., 2014) and years lived with a disability (Woolf, 2015; Hunter and Bierma-Zeinstra, 2019). Economic and societal costs are expected to rise worldwide during the coming years (Woolf, 2015; Ackerman et al., 2019). Direct costs include pharmacological and non-pharmacological interventions, surgeries, emergency room visits, and long-term care (Hunter et al., 2014; Xie et al., 2016). Among high-income countries, the estimated proportion of gross domestic product associated with osteoarthritis-related medical costs ranges from 1 to 2.5% (Hunter et al., 2014; Murphy et al., 2018). Personal costs include those incurred by loss of employment, premature retirement, and loss of income and personal savings (Gupta et al., 2005; Schofield et al., 2013, 2018; Woolf, 2015; Hunter and Bierma-Zeinstra, 2019), which increases the demand for sickness benefits and disability pensions (Woolf, 2015; Törmälehto et al., 2019). Further indirect costs result from factors such as absenteeism, presenteeism, reduced productivity, caregiver time, and premature mortality (Hunter et al., 2014; Xie et al., 2016).

The knee joint accounts for a substantial proportion of the global osteoarthritis burden (Hunter and Bierma-Zeinstra, 2019). Total Knee Arthroplasty (TKA) is an increasingly common surgical treatment for end-stage osteoarthritis of the knee among people in developed nations, including Australia. By 2030, demand for this surgery in Australia is expected to increase by 276% (from 2013 procedure figures), at a healthcare cost of approximately $1.38 billion (Ackerman et al., 2019). Similar increases are expected in other developed countries (Ackerman et al., 2019).

Total knee arthroplasty demonstrates high success rates in terms of objective medical outcomes, including survival and performance of prostheses, and low revision rates (Australian Orthopaedic Association National Joint Replacement Registry (AOANJRR), 2018; Evans et al., 2019). As such, surgeons often report high levels of satisfaction with postoperative outcomes (Jones et al., 2007). However, surgeon satisfaction and patient-rated satisfaction are not well correlated (Brokelman et al., 2003; Sasaki et al., 2014b). Up to 30% of patients report dissatisfaction with the outcomes of their TKA (Brander et al., 2003; Khatib et al., 2015; Alattas et al., 2017; Bierke and Petersen, 2017), despite the absence of radiological or observable physical complications, such as infection or problems with prostheses (Edwards et al., 2009; Bierke and Petersen, 2017). Patient dissatisfaction with TKA is higher when self-reported pain and functional outcomes do not improve (Baker et al., 2007; Bierke and Petersen, 2017) or recovery outcomes do not align with preoperative expectations (Bourne et al., 2010; Bryan et al., 2018; Swarup et al., 2018).

In order to understand patient dissatisfaction following TKA, it is important to consider the context in which people elect to have this “quality of life surgery” (Clark et al., 2004; Jones et al., 2007; Slover et al., 2012). For many patients, worsening pain is the most impactful symptom of osteoarthritis, and expectations about the potential for arthroplasty surgery to resolve pain strongly motivates the decision to proceed (Clark et al., 2004; Neogi, 2013). Other influential preoperative factors include symptom severity, level of functional disability, loss of independence, occupational, social and financial impacts and quality of life (Clark et al., 2004; Hall et al., 2008; Slover et al., 2012). Therefore, when postoperatively considering satisfaction with their TKA, patients may focus less on objective medical outcomes, and more on changes in pain and functional limitations, particularly as these factors affect participation in meaningful life activities (Baker et al., 2007; Alattas et al., 2017; Bierke and Petersen, 2017; Biggs et al., 2019). Although TKA generally leads to improvements in pain and function (Ayers et al., 2013; Ackerman et al., 2019), fully recovered patients often remain below general population norms (Jones et al., 2007). Furthermore, approximately 20–30% of patients report enduring pain for months to years after surgery (Beswick et al., 2012; Burns et al., 2015) and persistent functional impairments (Wylde et al., 2007; Bourne et al., 2010; Singh and Lewallen, 2014; Hirschmann et al., 2013). Affected patients are at higher risk of reporting diminished quality of life, difficulty performing activities of daily living, and poor physical and psychological health (Bourne et al., 2010; Jeffery et al., 2011; Bierke and Petersen, 2017; Bryan et al., 2018). If pain and function do not improve by 6 months, patients are more likely to be dissatisfied at 12 months (Scott et al., 2010) and experience increased depressive symptoms (Bryan et al., 2018). When dissatisfaction with the outcomes of TKA remains several years after surgery, impacts such as low range of motion and high levels of pain, anxiety, and depressive symptoms can persist for up to 13 years after surgery (Ali et al., 2014, 2017).

The high frequency of adverse patient-reported outcomes are not only important because of the role they play in patient dissatisfaction, but also because they reveal that a sizeable proportion of patients do not experience the intended quality of life benefits following TKA (Alattas et al., 2017). This has led to increased incorporation of patient-reported outcome measures (PROMs) in assessments of postoperative recovery. For example, the Australian Orthopaedic Association National Joint Replacement Registry (Australian Orthopaedic Association National Joint Replacement Registry (AOANJRR), 2018) recently launched a web-based pilot study in which joint surgery patients at 50 hospitals are assessed using PROMs of general health status, pain, function, comorbidities, and satisfaction. This will facilitate a better understanding of how patients experience recovery and restoration of quality of life following TKA. Furthermore, the preoperative assessment of specified variables presents the opportunity to prospectively identify risk factors for poor patient-centered health-related quality of life outcomes.

In this conceptual analysis, we present a case for a more comprehensive assessment of multidisciplinary preoperative risk factors for adverse patient outcomes following TKA, including patient-centered outcomes. We outline the limitations of current rehabilitation referral pathways, and explore how interdisciplinary collaboration and the adoption of precision medicine principles and methods may improve patient-centered outcomes and satisfaction among at-risk patients following TKA. Specifically, we focus on the development of a patient recovery outcomes prediction tool, based on multivariate machine-learning predictive models. This tool could be used to simplify the assessment of multidisciplinary factors outside of the usual practice of orthopedic surgeons, such as patient psychological characteristics, and improve the precision of rehabilitation referral decisions. We further explore how this tool could facilitate the development of individualized rehabilitation interventions, targeting modifiable patient characteristics and processes known to adversely affect TKA outcomes. In addition to providing a brief outline of the development process for this tool, we present a case for the importance and benefits of including a comprehensive assessment of psychological factors and processes that are known to predict patient recovery and quality of life outcomes.

## Referral Pathways and Rehabilitation Interventions

The restoration of patients’ quality of life, and physical and psychological functioning in relation to disability or illness are core goals of Rehabilitation Medicine (Royal Australasian College of Physicians [RACP], 2018; European Physical and Rehabilitation Medicine Bodies Alliance, 2018; Sciumè et al., 2018). Rehabilitation Medicine traditionally adopts a biopsychosocial multidisciplinary and multimodal approach to assessment and treatment (Negrini, 2019). Currently, medical rehabilitation is both a key facilitator of postoperative functional recovery following TKA (Papalia et al., 2013; Schilling et al., 2019) and an established component of a patient’s postoperative treatment plan. This places Rehabilitation Medicine in an important position to improve prognoses for patients at risk of poor postoperative outcomes. However, restoring patients to functional everyday life following surgery is challenging (Cai et al., 2018), and many factors can disrupt or impede the rehabilitation process.

Rehabilitation following TKA typically focuses on helping patients re-establish muscle strength, joint stability and neuromuscular control (Papalia et al., 2013), through engagement in sufficient levels of appropriate physical activity during recovery (Cai et al., 2018). Interventions are delivered in inpatient or outpatient settings, such as community-based facilities or a patient’s home (Papalia et al., 2013; Schilling et al., 2019). A large proportion of patients in Australia are referred to inpatient rehabilitation (Buhagiar et al., 2017; Schilling et al., 2019), despite growing evidence demonstrating this does not always lead to better TKA outcomes when not clinically indicated (Naylor et al., 2017; Padgett et al., 2018; Buhagiar et al., 2019; Schilling et al., 2019). For example, Buhagiar et al. (2017) assessed post-TKA outcomes among patients who had completed home or inpatient programs, excluding those with comorbidities, who lacked social support, or were unable to perform rehabilitation activities without assistance. Despite reporting higher levels of satisfaction, patients who completed inpatient rehabilitation demonstrated no differences in mobility, function, or quality of life outcomes compared to those who completed home-based rehabilitation. In other studies, outpatient and home-based interventions have been associated with better recovery outcomes (Papalia et al., 2013) and patient satisfaction than inpatient rehabilitation (Crawford et al., 2015).

Individual patient characteristics may play an important role in the degree of rehabilitative supervision required for optimal recovery (Papalia et al., 2013), highlighting the importance of considering these when referring patients to rehabilitation services. While a select group of relevant patient indicators, such as age, social support and comorbidities can play a role in rehabilitation referral destination, the lack of standardized rehabilitation referral pathways for TKA patients in Australia has been identified as a concern (Rehabilitation Medicine Society of Australia and New Zealand [RMSANZ], 2018; Royal Australasian College of Surgeons [RACS], 2018). Existing rehabilitation referral practices vary widely between hospitals and surgeons (Royal Australasian College of Surgeons [RACS], 2018), and can be influenced by non-clinical factors, such as patient expectations, hospital business models, and insurance-related factors (Rehabilitation Medicine Society of Australia and New Zealand [RMSANZ], 2018; Royal Australasian College of Surgeons [RACS], 2018).

These referral processes present a concerning situation in which some patients may not receive the most appropriate form of rehabilitation for their specific circumstances. At best, this may result in an inefficient allocation of healthcare resources and funding (Buhagiar et al., 2017; Royal Australasian College of Surgeons [RACS], 2018). At worst, this process may be a contributing factor to suboptimal recovery, including poor patient-centered health-related quality of life outcomes and dissatisfaction. With the goal of improving current referral pathways for rehabilitation following joint surgery and improving patient safety and recovery outcomes, the Rehabilitation Medicine Society of Australia and New Zealand [RMSANZ] (2018) recommended that individual patient risk factors such as age, health status, baseline levels of knee-related physical functioning, and social support status should form the basis of the type of rehabilitation patients receive. While this recommendation represents a critical step toward ensuring rehabilitation referral decisions are more appropriately aligned with patients’ specific risk factors, it remains limited by the fact that it guides only the decision about *where* to refer patients for their rehabilitation, without reference to *what* type of rehabilitation program might best suit their individual needs.

It has been recommended that optimal rehabilitation outcomes for individual patients are more likely to be achieved through the provision of needs-specific interventions that are appropriately timed and -dosed (Negrini, 2019). There is no identified single predictor of adverse patient-centered outcomes following TKA (Jones et al., 2007). Rather, the literature suggests that recovery trajectories are influenced by a broad range of factors that are not necessarily part of routine assessment, referral, or rehabilitative intervention practices for medical professionals involved in TKA care. For example, general psychological distress, which occurs at high rates among joint replacement patients (30%, Ayers et al., 2004), is predictive of the degree of satisfaction, functional recovery, and pain experienced by patients following TKA (e.g., Scott et al., 2010; Paulsen et al., 2011; Khatib et al., 2015). Systematic review findings suggest that modifiable psychological variables are significantly more predictive of postoperative pain than other clinical indicators, such as perioperative and biomechanical variables (Lewis et al., 2015). Rehabilitation interventions targeting modifiable psychological risk factors may lead to improved postoperative outcomes for vulnerable patients, particularly if interventions target the individual risk profile for each patient (Bierke and Petersen, 2017). However, translating the broad and complex range of psychological predictors into clinically effective, meaningful and manageable assessment, referral and practice methodologies has the potential to be complex. This is particularly the case since many of these factors fall outside of the usual purview of surgeons and rehabilitation providers (Negrini, 2019; Pattichis et al., 2019). Determining which variables to assess and the relative impacts of the vast range of preoperative psychological factors that may affect each patient’s post-TKA recovery is difficult to achieve using existing research evidence alone. As such, it is likely that many patients are not referred to the rehabilitation they need because relevant factors are not screened prior to surgery.

## Interdisciplinary Precision Medicine and the Prediction of Patient Recovery Trajectories

We propose an innovative approach to address these referral and rehabilitative intervention challenges, that could facilitate a considerable reduction in the rates of patient-reported post-TKA dissatisfaction and adverse outcomes. Such an approach would involve the adoption of precision medicine principles that move beyond basing referral and treatment decisions on limited clinical indicators and averaged patient data, toward comprehensive interdisciplinary models of assessment and individualized care. Synthesis of interdisciplinary knowledge pertaining to identified predictors of TKA patient-centerd outcomes would guide the development of advanced machine-learning multivariate models. The proposed multivariate model would predict the probability of particular individual patient recovery trajectories and postoperative outcomes, based on a range of personal characteristics (Sanchez-Santos et al., 2018), such as medical, demographic, social, lifestyle, environmental, and psychological factors (Flynn et al., 2019). This could provide important predictive insights into the potential impacts of unique combinations of these factors on an individual’s recovery (Flynn et al., 2019; Pattichis et al., 2019). The clinician-driven use of a predictive tool of this nature could simplify outcome predictor assessment and referral pathway decision-making for orthopedic surgeons, as well as guide and improve individual patient recovery through the delivery of customized and individualized interventions targeting modifiable outcome predictors (Pattichis et al., 2019).

**FIGURE 1:**
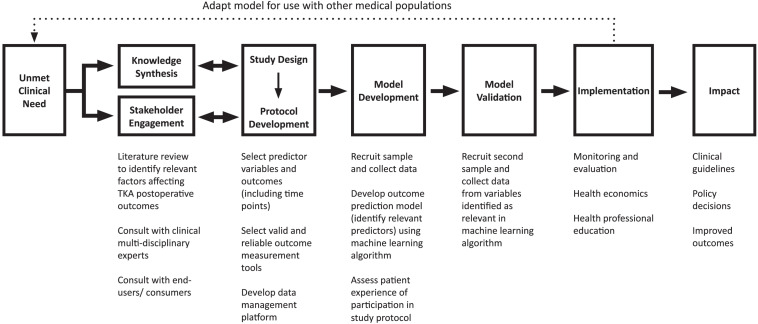
Multivariate post-TKA outcomes prediction tool development process.

In order for our proposed model to have optimum predictive power, we argue that it must draw on research and expertise from a broad range of relevant disciplines, including but not limited to orthopedic surgery, rehabilitation medicine, psychology, physiotherapy, and exercise physiology. Furthermore, the development and validation of powerful predictive algorithms requires interdisciplinary collaboration with experts in fields like engineering and data science. While the development of such a tool is a complex and time-intensive process, we believe that a thorough and intensive investment at the research and evaluation level will simplify and enhance interdisciplinary collaboration and knowledge-sharing in efficient and practical ways. This has potential advantages for both healthcare practitioners and patients. While a detailed description of the methodological processes involved in the development of the proposed tool are beyond the scope of this conceptual analysis and will be presented in future publications, Figure 1 presents an outline of key stages.

The development of multivariate prediction models of patient postoperative recovery outcomes is gaining prominence. A number of existing models have examined predictors of patient-centered TKA outcomes, such as satisfaction (van Onsem et al., 2016; Kunze et al., 2019), pain (Dowsey et al., 2016; Sanchez-Santos et al., 2018), and function (Dowsey et al., 2016; Sanchez-Santos et al., 2018; Kunze et al., 2019), as well as discharge destination (Hansen et al., 2015) and outcomes of osteoarthritis rehabilitation interventions (Weigl et al., 2006). These models have been used to aid decisions regarding whether surgery will likely achieve the desired outcomes for particular individuals (Dowsey et al., 2016; van Onsem et al., 2016) and establish preoperative expectations for individual recovery outcomes (Sanchez-Santos et al., 2018). However, all of these TKA models have proposed a linear model, i.e., the outcome measure is assumed to be predicted by a weighted sum of the input factors. In some cases, the output is thresholded using a Logistic model (Weigl et al., 2006; Dowsey et al., 2016; Kunze et al., 2019) while in others a continuous output is predicted (van Onsem et al., 2016; Sanchez-Santos et al., 2018; Kunze et al., 2019). In either case, these linear models are not able to capture complex relationships between the predictors. For example, a person’s psychological response to pain may impact their recovery trajectory, but only when their reported pain score is also high. In this case, a model then should only include a weighting for psychological response to pain when pain is above some threshold, and exclude it otherwise. A linear model is not able to do this.

The development of the type of multivariate outcome prediction tool we are proposing requires statistical evaluation models that can accurately predict recovery based on an individual patient’s status across a broad range of factors (Flynn et al., 2019). In order to facilitate rehabilitative interventions that precisely target an individual patient’s specific combinations of modifiable risk factors, a method of modeling that is better suited to this goal is required. This can be achieved using more sophisticated, non-linear, machine-learning models and temporal validation. Temporal validation involves the development of a model based on data collected from an initial TKA patient sample, and subsequent validation on a second sample recruited at a later time point, where the same data has been collected. To date, only one of the existing TKA prediction models has been externally validated (Sanchez-Santos et al., 2018).

## The Role of Modifiable Psychological Factors

### Psychological Risk and Resilience Factors

Psychological characteristics relate to an individual’s cognitive, emotional, and behavioral responses to their experiences. These factors play an important role in one’s capacity to cope with and adjust to acute and chronic stressors (Sturgeon and Zautra, 2013; Goubert and Trompetter, 2017). In the case of TKA, adjustment and recovery depend on a patient’s ability to restore function and reduce disability following surgery (Goubert and Trompetter, 2017). Certain psychological factors can function as vulnerabilities that hinder postoperative recovery and rehabilitation progress (Sturgeon and Zautra, 2013). Others can function protectively, leading to higher levels of adjustment and resilience (Goubert and Trompetter, 2017).

The limited attention given to modifiable psychological factors in post-TKA rehabilitative referral and intervention practices, relative to the prominence and importance of these factors reported in the literature, represents a problematic research-to-practice gap which may contribute to adverse patient outcomes. Multivariate machine-learning prediction models provide a potential mechanism through which to link modifiable psychological variables and TKA outcomes to practical rehabilitation practices. Such models could facilitate the expansion of rehabilitative interventions beyond biologic models and toward the biopsychosocial model, which explains models of human behavior and recovery from illness more accurately than biologic models alone.

Existing TKA prediction models (e.g., Tan et al., 2014; Dowsey et al., 2016; Sanchez-Santos et al., 2018; Kunze et al., 2019) have largely ignored psychological factors, focusing instead on medical and non-modifiable demographic predictors of adverse outcome. Models that have incorporated psychological factors have assessed “mental health” using general measures (e.g., Dowsey et al., 2016) or single-item measures that do not distinguish between depression and anxiety (e.g., van Onsem et al., 2016; Sanchez-Santos et al., 2018). These measures lack the precision to demonstrate the potential differential impacts of varying psychological factors (Alattas et al., 2017) or meaningfully guide rehabilitation interventions targeted at specific psychological symptoms or processes. Where existing models do include a more thorough assessment of psychological factors (e.g., van Onsem et al., 2016), these account for the majority of modifiable predictors assessed in the model. This highlights the importance of considering the role of psychological variables in post-TKA outcome prediction, as they are potential targets of interventions that could enhance recovery. It is important to note that these models focus on psychological vulnerability factors, and that no measures of psychological resilience are included.

We propose that an outcomes prediction model should include a comprehensive assessment of relevant psychological risk and resilience factors, utilizing valid and reliable assessment measures for each construct under investigation. Measures should preferably have demonstrated validation with TKA or related samples (e.g., osteoarthritis or chronic pain patients). It is important that care is taken to select measures that keep participant assessment burden to a minimum, and that any measures that do not predict outcome variance are removed from clinical versions of the predictive tool.

We provide an outline of the predictive and potential roles of several modifiable psychological vulnerability (Table 1) and resilience factors (Table 2) in TKA recovery, in order to demonstrate the importance of including these in a multivariate recovery prediction model.

**TABLE 1:** Psychological vulnerability factors and associated adverse postoperative TKA outcomes.

Vulnerability factor	TKA outcomes
**Depression**	– Worse knee stiffness and disability – Poorer functional recovery – Higher pain severity – Increased risk of chronic pain – Smaller improvements in pain – Longer post-operative hospital stay – Poorer motivation and adherence to rehabilitation intervention – Poorer expectations of rehabilitation outcomes – Poorer quality of life – Higher 1-year mortality rates (elderly patients)
**Anxiety**	– Worse knee disability – Poorer functional recovery and physical functioning – Higher severity post-operative pain (immediately following surgery) – Higher pain severity – Increased risk of chronic pain – Increased likelihood of avoidance behaviors – Some evidence of longer-term impact (up to 10 years), while other evidence suggests effects diminish after 1 year
**Pain catastrophising**	– Higher severity post-operative pain (immediately following surgery) – Worse pain throughout the recovery process – Increased risk of chronic pain (years after surgery) – Smaller improvements in pain – Poorer knee function – Increased functional disability – Increased likelihood of avoidance behaviors

**TABLE 2:** Potential psychological resilience factors and their demonstrated protective impacts in related populations (e.g., other orthopedic postoperative patients, chronic pain patients, and patients with OA).

Protective factor	Health and wellbeing outcomes
**Resilience**	– Better post-orthopedic surgery patient- and surgeon-rated satisfaction – Higher post-rehabilitation functional independence – Buffers impact of pain intensity on functional disability – Buffers maladaptive cognitive and emotional responses to pain – Lower levels of pain-related disability – Lower 10-year morbidity rates – Better mental health-related quality of life – Fewer anxiety and depressive symptoms – Lower rates of pain catastrophising – Less pain-related fear
**Committed action**	– Better pain acceptance – Fewer depressive symptoms – Better physical functioning – Better social functioning – Better mental health – Better general health – Higher rates of vitality – Reduced behavioral and experiential avoidance – Reduced rigid adherence to cognitive rules, beliefs, and emotions – Capacity for flexible engagement in valued life activities, potentially increasing quality of life – Capacity for flexible persistence and responsivity to direct experiences, potentially improving effective rehabilitation engagement

#### Psychological Vulnerability Factors

In TKA and orthopedic surgery research, depression, and anxiety are generally assessed using PROMs, which provide estimates of depressive and anxiety symptom severity rather than formal clinical diagnoses. Importantly, predictive relationships between these factors and TKA outcomes have been observed, indicating that anxiety and depressive symptoms can impact outcomes even when they do not meet clinical diagnostic thresholds, and that PROMs can be used to effectively screen patients (Bistolfi et al., 2017). For the purpose of this paper, the terms *depression* and *anxiety* refer to the presence of significant levels of these symptoms among patient samples. It is important to note that, while anxiety and depression can occur comorbidly, they are distinct constructs that should be assessed separately in risk prediction models in order to detect their unique impacts for individualized intervention planning (Alattas et al., 2017).

##### Depression

Depressive symptoms are common among patients with osteoarthritis (19.9%: Stubbs et al., 2016) and those undergoing arthroplasty (10–33%: Browne et al., 2014; Ali et al., 2017; Bistolfi et al., 2017). Symptoms include life-interfering disruptions to mood (e.g., increased negative affect, diminished positive affect, and feelings of worthlessness), cognitive functioning (e.g., difficulty concentrating and making decisions, negative expectations and perceptions of experiences) and behavior (e.g., physical slowing down and reduced motivation to engage in behavioral activity) (American Psychiatric Association, 2013). Patients demonstrating preoperative depressive symptoms appear at greater risk of adverse outcomes following TKA and other orthopedic surgeries (Khatib et al., 2015; Lewis et al., 2015; Lungu et al., 2016; Alattas et al., 2017).

Several systematic reviews and meta-analyses have demonstrated associations between preoperative depression and postoperative pain and functional outcomes following TKA. For example, higher preoperative depressive symptoms have demonstrated predictive value in relation to higher levels of knee disability and stiffness, worse pain and function, chronic pain, and smaller improvements in pain and function (Khatib et al., 2015; Lewis et al., 2015; Lungu et al., 2016; Alattas et al., 2017). While some studies have demonstrated relationships between depressive symptoms and adverse pain and function outcomes up to a year following TKA (e.g., Hanusch et al., 2014; Singh and Lewallen, 2014; Lewis et al., 2015; Alattas et al., 2017; Wylde et al., 2017), others report impacts that endure for many years (e.g., Brander et al., 2007; Khatib et al., 2015; Lungu et al., 2016). In one systematic review and meta-analysis, preoperative depression explained 10% of the variance in knee function scores 5 years post-TKA, and the relationship between depression and poor functional recovery was predictive of postoperative pain at various stages of longer-term follow-up (Khatib et al., 2015).

Other outcomes associated with pre-operative depression include longer hospital stays after surgery (March et al., 2018), poorer engagement in postoperative physiotherapy rehabilitation (Jack et al., 2010), and lower motivation, outcome expectations, and adherence to inpatient rehabilitation (Tung et al., 2013). For elderly orthopedic surgery patients, preoperative depressive symptomatology appears to augment the risk of adverse outcomes. For example, older women with depression who had completed rehabilitation following orthopedic surgery were at particular risk of diminished quality of life (Tung et al., 2013). Furthermore, 1-year mortality rates for severely depressed elderly patients discharged from inpatient rehabilitation after lower-limb surgery were four times higher than the rates among elderly patients who were not depressed (Guerini et al., 2010).

Depressive symptoms may disrupt recovery processes in many ways. Behaviorally, the physical slowing and diminished motivational components of depression may affect a patient’s capacity to engage in rehabilitation tasks and appropriate levels of daily physical activity (Tung et al., 2013). Reduced behavioral activation also likely limits a patient’s contact with positively reinforcing consequences of exercise that might otherwise contribute to sustained engagement. Cognitive impacts, such as negative expectations and/or perceptions, may also affect engagement in restorative physical activities or skew a patient’s perception of recovery cues. These factors may affect the restoration of quality of life through the limitation of engagement in meaningful and purposeful life activities (Smith and Zautra, 2004). In relation to mood symptoms, the infrequent experience of positive emotions and frequent experience of negative emotions may influence recovery by restricting an individual’s attentional focus toward negative recovery cues. Such attentional “narrowing” may limit the range of coping behaviors available to a patient when dealing with health-related stressors (Waugh and Fredrickson, 2006; Goubert and Trompetter, 2017).

Depressive symptoms can be modifiable through the implementation of a range of cost-effective evidence-based interventions, including cognitive-behavioral therapies (CBT), interpersonal therapy, mindfulness-based cognitive therapy, problem-solving therapy, acceptance and commitment therapy (ACT), psychoeducation and antidepressant medication (Australian Psychological Society [APS], 2018). The identification of patients with high severity preoperative depressive symptoms would allow targeted interventions to be implemented to benefit postoperative recovery.

##### Anxiety

Anxiety is a broad term used to describe a range of fear-related symptoms across a spectrum of severity. Individuals with anxiety may experience cognitive (e.g., worry and threat-oriented attentional focus), emotional (e.g., fear), physiological (autonomic arousal, including elevated heart rate, and respiration), and/or behavioral symptoms (e.g., avoidance) (American Psychiatric Association, 2013). Anxiety is a manifestation of the normal human fear response but may be excessive and maladaptive in certain circumstances (Evans et al., 2012), and is often directed toward anticipated future experiences (Leeuw et al., 2007). Experiencing anxiety specifically in anticipation of upcoming elective surgery is almost universal, with prevalence rates as high as 92.6% (Aust et al., 2018). However, a substantial proportion of elective surgery patients (40.5%) experience preoperative anxiety that is more severe (Aust et al., 2018). In samples of patients undergoing TKA, comorbid anxiety rates of 14.5% have been reported (Bierke and Petersen, 2017). Compared with non-anxious patients, those reporting high levels of preoperative anxiety are almost twice as likely to report dissatisfaction with outcomes 6 months after surgery (Bierke and Petersen, 2017), and utilize healthcare services at higher frequencies for up to 1 year after TKA (Brander et al., 2003).

Preoperative anxiety is associated with adverse outcomes following TKA, including greater risk of experiencing chronic pain (Bierke and Petersen, 2017), higher pain severity and lower function (Brander et al., 2003; Wylde et al., 2012, 2017; Hanusch et al., 2014; Sanchez-Santos et al., 2018).

Systematic reviews and meta-analyses demonstrate a predictive relationship between preoperative anxiety and TKA outcomes. For example, Alattas et al. (2017) found that high preoperative anxiety is a predictive factor for postoperative knee disability, functional limitations, and higher pain severity. Furthermore, along with preoperative pain and function, anxiety predicts poor outcomes up to 10 years after surgery. In another review and meta-analysis of prospective observational TKA outcome studies (Khatib et al., 2015), a higher severity of preoperative anxiety predicted worse postoperative pain, poorer functional recovery, and low levels of physical functioning. Postoperative pain was more severe for highly anxious patients than non-anxious patients at all follow-up times ranging from 6 months to 5 years (average 14 months). Lungu et al. (2016) also found preoperative anxiety or depressive symptoms predicted worse pain and function, and lower levels of knee function improvement. However, they also concluded that psychosocial factors demonstrated less consistent relationships with pain and function outcomes than clinical factors, such as preoperative pain and function. Similarly, other studies have reported weaker associations between TKA outcomes and high levels of anxiety and chronic pain, than for depression and pain catastrophising. Longitudinal studies have also reported that the effects of anxiety on TKA outcomes 1 year after surgery are no longer evident at 5 years (Brander et al., 2007; Wylde et al., 2017).

The Fear-Avoidance Model (Leeuw et al., 2007; Crombez et al., 2012), provides a useful framework to understand the relationship between anxiety and adverse postoperative outcomes. According to the model, the experience of pain activates normal threat response processes leading to avoidance behaviors. While these responses can be adaptive, if these responses are rigidly adhered to when pain is not signaling threat, these can instead lead to maladaptive longer-term pain-related coping patterns (Sturgeon and Zautra, 2013). Following TKA, anxiety may predispose patients to avoid stimuli, activities, sensations or situations that they fear will exacerbate their condition (Theunissen et al., 2012), such as musculoskeletal physiotherapy rehabilitation programs that may cause pain or re-injury (Jack et al., 2010). This can result in longer-term adverse outcomes such as physical deconditioning, comorbidities, loss of function and muscle tone, and enduring chronic pain (Bortz, 1984; Sullivan et al., 2009; Powell et al., 2013). Avoidance behaviors, therefore, can ultimately lead to augmentation of the outcome that an individual is seeking to diminish (DeGaetano et al., 2016), such as worse pain intensity and longer-term disability (Goubert and Trompetter, 2017), resulting in further avoidance and the perpetuation of a detrimental fear-avoidance behavioral cycle (Theunissen et al., 2012).

Anxiety symptoms can be modifiable through evidence-based interventions such as CBT, ACT, mindfulness-based cognitive therapy, mindfulness-based stress reduction, and psychoeducation (Australian Psychological Society [APS], 2018). The preoperative assessment of anxiety for patients undergoing TKA would allow for the identification of at-risk patients, and the implementation of intervention strategies that could mitigate the adverse impacts of these symptoms.

##### Pain catastrophising

Pain catastrophising is a psychological construct that reflects problematic cognitive and emotional responses to pain or expected pain (Sullivan et al., 2009). Specifically, it involves perceptions and interpretations of pain and pain-related stimuli that are magnified and overly threat-focused, feelings of helplessness in response to pain, and the belief that nothing can be done to control or relieve pain (Sullivan, 2009; Sturgeon and Zautra, 2013; Birch et al., 2017). Patients who demonstrate high pain catastrophising tend to focus excessively on pain-related sensations and thoughts, and have difficulty shifting their attention away from these internal cues.

Nearly a third of patients undergoing TKA exhibit pain catastrophising (Bierke and Petersen, 2017). This likely affects their reporting of pain severity and duration (Sullivan et al., 2009; Birch et al., 2017), and recovery trajectories. March et al. (2018) propose that process factors that reflect a person’s psychological coping skills, such as pain catastrophising, may be more sensitive than symptom-based measures in detecting who will be at risk of poor recovery after TKA.

Preoperatively identified pain catastrophising is strongly related to patient-reported postoperative short-term pain outcomes. This has been observed in patients following TKA (Vissers et al., 2012; Burns et al., 2015), and is independently predictive of pain severity 6 weeks after surgery (Sullivan et al., 2009). Pain catastrophising has been found to be a significant reliable predictor of chronic pain, lower pain improvement, and greater pain intensity three or more months after TKA (Vissers et al., 2012; Burns et al., 2015; Khatib et al., 2015; Lungu et al., 2016). Burns et al. (2015) observed no effect of follow-up time on the strength of this relationship. In a multivariate meta-analysis of factors associated with chronic pain after TKA (Lewis et al., 2015), preoperative pain catastrophising was one of the strongest predictors, an effect that persisted for many years following surgery.

Because pain catastrophising increases a person’s perception that their pain is an indicator of threat or harm, this may also trigger avoidance behavior (Sturgeon and Zautra, 2013), contributing to worse postoperative outcomes. By limiting an individual’s exposure to pain and pain-related stimuli, avoidance can result in failure to extinguish pain-related fears (Leeuw et al., 2007; Powell et al., 2013), reduced efforts to develop more diverse and flexible pain-related coping resources (internal or external), and decreased likelihood of ceasing ineffective behavioral coping mechanisms in response to pain (Sturgeon and Zautra, 2013). Furthermore, when patients are unable to shift their attention away from pain and related experiences after surgery, they may experience difficulty attending to broader experiences, including more adaptive emotions, cognitions, or behaviors. This may impede a patient’s capacity to experience positive reinforcement and motivation in relation to positive recovery cues. Together, these processes may disrupt a patient’s capacity to restore function and behaviors that enhance quality of life. This could explain relationships between pain catastrophising and function-related postoperative TKA outcomes, including longer time taken to achieve a 90- degree bend (Kendell et al., 2001), higher levels of functional disability (Lungu et al., 2016) and poorer knee function up to 1 year after surgery (Bierke and Petersen, 2017; Wylde et al., 2017).

It has been proposed that, because chronic pain is a pre-existing clinical factor for most patients undergoing musculoskeletal surgery, pain catastrophising may exert its influence by hindering recovery from pre-existing chronic pain rather than exacerbating new pain resulting from surgery (Theunissen et al., 2012). This has important implications for the timing of rehabilitative interventions targeted at modifying pain catastrophising, suggesting that earlier management of these symptoms may be optimal.

Certain intervention strategies may be beneficial in modifying pain catastrophising among TKA patients (Gibson and Sabo, 2018). For example, CBT delivered during rehabilitation following TKA has led to reduced pain catastrophising and improved pain and knee function among patients with high levels of kinesiophobia. These outcomes were demonstrated 6 months after surgery and were superior to standard rehabilitative care (Cai et al., 2018). Other studies have demonstrated the efficacy of physiotherapy interventions that include a CBT component (Gibson and Sabo, 2018). Other interventions that have demonstrated efficacy in modifying pain catastrophising among surgical patients include psychoeducation, physiotherapy with a neuroscience component, and the immediate postoperative administration of a standard dose of pregabalin (Lyrica) (Gibson and Sabo, 2018).

It should be noted that there may be collinearity between anxiety, depression, and pain catastrophising (Burns et al., 2015). This would not pose a problem for a multivariable non-linear machine-learning prediction tool. In such a model, risk factors can be assessed at the individual item level, which allows for the assessment of key elements of each of these conditions that predict poor outcomes. Not only does this approach allow for the simplified assessment of the complex range of psychological symptoms and processes that may affect post-TKA recovery outcomes, but it also may facilitate even more precise and efficient individualized intervention strategies targeted only at the aspects of each factor that are impacting outcomes. For example, a CBT rehabilitative intervention might focus on only the cognitive aspects of depression and anxiety, if these emerged as the most impactful modifiable psychological factors for a particular patient.

#### Psychological Resilience Factors

##### Resilience

Resilience is a broad term that encompasses a range of coping resources that individuals draw on to deal with adversity. Generally, resilience refers to one’s ability to function effectively when experiencing internal distress or stressful circumstances (Connor and Davidson, 2003; Karoly and Ruehlman, 2006). When coping with TKA and postoperative recovery, resilient individuals may be able to utilize cognitive, emotional, and/or behavioral resources that maintain their connection with personal values, restore a sense of equilibrium, and optimize health-related quality of life outcomes (Sturgeon and Zautra, 2013). This may include the ability to bounce back from stress (Smith et al., 2010), self-efficacy, effective engagement with social support networks, perseverance, frequent experience of positive emotions, optimism, acceptance, and psychological flexibility processes such as committed action (Viggers and Caltabiano, 2012; Goubert and Trompetter, 2017).

While psychological vulnerabilities demonstrate predictive validity with respect to TKA outcomes, focusing only on maladaptive coping processes and patient risk indicators provides an incomplete understanding of the factors affecting patient recovery. For example, while some studies have shown that the postoperative resolution of knee-related disability is associated with the subsequent resolution of depressive symptoms among preoperatively depressed patients (Blackburn et al., 2012; Fehring et al., 2018), others demonstrated no improvement and little variation in depressive symptoms over a 12-month period following successful TKA (Bistolfi et al., 2017). Such conflicting findings might be indicative of the influence of unassessed resilience mechanisms on patient recovery trajectories, enhancing adaption following surgery and buffering the impact of psychological vulnerabilities for some patients (Sturgeon and Zautra, 2013). Inclusion of resilience processes into predictive models may help to explain good postoperative outcomes in the context of poor preoperative psychological and clinical symptoms, clarifying how resilience factors may operate concurrently with risk factors. This represents a conceptual shift from the assessment of pathology to understanding personal characteristics that allow an individual to thrive under challenge (Connor and Davidson, 2003). Assessment of these factors would facilitate a more comprehensive approach to predicting individual recovery trajectories following TKA and could provide further targets for intervention.

Resilience may buffer the impact of pain intensity on functional disability. Elliott et al. (2014) showed approximately one-third of a cohort of individuals with high-intensity pain were highly resilient and had low levels of pain-related disability. Individuals demonstrating these characteristics had substantially lower 10-year morbidity rates than non-resilient people. Resilience is also associated with better outcomes in patients suffering chronic pain, such as with osteoarthritis (Goubert and Trompetter, 2017). For example, chronic pain patients demonstrating higher resilience reported better mental health-related quality of life and lower levels of anxiety and depression (Viggers and Caltabiano, 2012). Resilience was more strongly associated with mental health outcomes than pain severity or perceived physical quality of life, demonstrating the potential facilitation of quality of life restoration despite the presence of health-related stressors. Resilience can also buffer maladaptive cognitive and emotional responses to pain: highly resilient people tend to report lower levels of pain catastrophising and pain-related fear (Karoly and Ruehlman, 2006).

While there is little information about the role of resilience in post-TKA recovery specifically, there exists important findings from other orthopedic surgical procedures and rehabilitation groups. For example, preoperative resilience predicted postoperative surgeon- and patient-reported outcomes up to 2 years after total shoulder arthroplasty surgery (Tokish et al., 2017), and was associated with higher levels of functional independence following a postoperative rehabilitation program among hip fracture patients (Sciumè et al., 2018). Viggers and Caltabiano (2012) propose that resilience may allow patients to persist with rehabilitation activities by broadening their attention toward a range of adaptive resources.

Collectively, these findings have important implications for the potential role of resilience processes in relation to postoperative rehabilitation engagement and recovery outcomes. Rehabilitation interventions targeted at enhancing modifiable resilience skills and processes may facilitate restoration of quality of life and reduction of functional disability even when, for example, pain reduction is not possible following TKA. Such interventions could have beneficial impacts on patient mental health and satisfaction ratings (Viggers and Caltabiano, 2012). This further reinforces the importance of assessing patient-centered outcomes that reflect quality of life, such as satisfaction and functional disability. Sciumè et al. (2018) propose that there is anecdotal evidence of the use of cognitive behavioral interventions with rehabilitation populations, and that efficacy in relation to resilience-building should be formally assessed.

##### Committed action

Committed action is a potential resilience factor. It is an important component of the Psychological Flexibility Model, which assumes that adverse internal experiences (e.g., pain, anxiety, and psychological distress) are normal and nonpathological, but exert adverse impacts through their capacity to impede an individual’s behavioral commitment to their personally-held values (Hayes et al., 1999; Bond et al., 2011). People who demonstrate psychological flexibility are less likely to rigidly adhere to cognitive rules or respond with avoidance in the presence of discomfort (physical or emotional). They demonstrate willingness to connect mindfully and openly with the full range of their cognitive, emotional, and sensory experiences and respond in ways that maintain consideration of their broader values and goals (McCracken, 2013; Scott and McCracken, 2015). It has been proposed that the Psychological Flexibility Model complements the fear-avoidance model (McCracken, 2013).

Specifically, committed action reflects the ability to flexibly pursue values-related life goals in the presence of discomfort (McCracken, 2013), using a wide range of effective actions (Trindade et al., 2018). In the case of TKA recovery, discomfort may include experiences such as pain, functional difficulties, or anxiety. Two key components of this “flexible persistence” skill (McCracken, 2013) may be of importance to postoperative recovery outcomes. Firstly, patients who are able to cope with uncomfortable experiences without engaging in avoidant behaviors may be more likely to persist with rehabilitation (and other) activities that restore function. Secondly, the ability to flexibly pursue one’s goals reflects the ability to choose behaviors that are responsive to direct experiences (McCracken, 2013). This means that individuals will be more aware of the workability of their coping strategies in relation to their values and goals, allowing them to adapt their responses in relation to their effectiveness, rather than rigidly adhering to unhelpful cognitive rules, beliefs, or emotions (McCracken, 2013). This is in contrast with pain catastrophising, where ruminative fusion with unhelpful cognitions and emotional responses are definitive components.

Currently, there is no research regarding the potential for committed action skills to improve patient outcomes following TKA. However, findings related to other conditions suggest its potential relevance to patient outcomes (McCracken et al., 2015; Trindade et al., 2018). For example, committed action has been shown to mediate the relationship between experiential avoidance and depression among breast cancer patients (Trindade et al., 2018), and has demonstrated a negative correlation with depression, and positive correlations with pain acceptance, physical functioning, social functioning, mental health, vitality, and general health among chronic pain patients (McCracken et al., 2015). This provides support for this process as a resilience factor, and suggests that strengthening committed action skills may buffer individuals from poor recovery outcomes.

Committed action can be enhanced in outpatient settings, using cognitive behavioral therapies such as ACT. Rather than focusing on the goal of reducing symptoms, such as pain or anxiety, the focus of ACT is on restoring a person’s capacity to engage in activities associated with valued living (Scott and McCracken, 2015). This is consistent with patient-centered post-TKA rehabilitative goals of restoring quality of life and enhancing functional participation. There is evidence that ACT interventions are effective in eliciting adaptive behavior change over the long-term (Hayes et al., 2012) and, as such, committed action may be a useful target of resilience-building rehabilitative interventions following TKA.

## Discussion

Adverse patient-reported outcomes following TKA occur at rates greater than objectively measured poor medical outcomes. This suggests that routine assessment of PROMs could provide a strong foundation on which to better understand and cater to individual patient needs. We see an opportunity to use interdisciplinary precision medicine methods to review and refine current rehabilitation referral and intervention pathways following TKA, with the goal of optimizing patient-centered outcomes. We propose the development of a non-linear, multivariate, machine-learning TKA recovery prediction model, drawing on the research base and expertise of multiple disciplines. This would involve the preoperative assessment of individual modifiable factors that impact individual recovery trajectories to facilitate the development of individualized multidisciplinary rehabilitation plans based on patients’ unique risk and resilience profiles. This model would simplify the assessment of a vast range of individual variables, including complex psychological risk and resilience factors, in a way that provides a clinically meaningful understanding of how they may impede or enhance individual patient recovery following TKA. This approach could serve to bridge the gap between existing research and clinical practice for orthopedic surgeons and multidisciplinary rehabilitation providers, who would have access to simplified methods of improving the precision of referral and intervention decisions, using important interdisciplinary information that is outside of their usual purview. This would represent a major shift in the way post-TKA rehabilitation is delivered, providing the potential for a greater degree of interdisciplinary collaboration, precision in interventions, and more efficient service provision and resource allocation.

Existing TKA outcome prediction models represent a positive step toward facilitating a stronger understanding of the many predictors of adverse patient postoperative outcomes. However, we propose that the range of modifiable psychological risk and resilience predictors that are prominent in these models are too limited, and currently are not routinely assessed in clinical practice. We have outlined the role of depression, anxiety, and pain catastrophising as risk factors for poor pain, function, and other quality of life outcomes among a proportion of patients following TKA. Furthermore, we have provided an argument for the potential importance of resilience-related patient characteristics, including committed action, in buffering patients from adverse postoperative outcomes. We propose that the assessment of a range of cognitive, emotional, and behavioral vulnerabilities and protective factors will facilitate a better understanding of the unique ways these psychological variables may interact to affect individual recovery trajectories. This will allow rehabilitation interventions to fully capitalize on the power of precision medicine techniques by focusing on modifiable cognitive, emotional, and behavioral factors relevant to individual patients. This could be key to resolving the significant rates of patient dissatisfaction observed following TKA.

Our interdisciplinary research group is currently in the process of developing a multivariate TKA outcomes prediction tool aligned with the objectives outlined in this conceptual analysis. We aim to predict postoperative recovery outcomes for patients undergoing TKA, based on pre-operative assessment of the range of factors presented here. Our model adopts a non-linear machine-learning approach that allows better handling of the large number of predictor variables we are assessing. We anticipate this model will allow for greater precision in the prediction of individual patient recovery trajectories, as well as highlighting modifiable targets for individualized rehabilitation interventions with greater specificity. We aim to develop and externally validate the model with separate patient samples. To date, only one of the existing TKA prediction models has been successfully validated externally (Sanchez-Santos et al., 2018). To our knowledge, there are no existing prediction tools meeting the criteria outlined in this conceptual analysis in routine clinical use to support and simplify musculoskeletal surgery rehabilitation referral decisions or individualized intervention delivery. Beyond TKA, we propose to validate our prediction model with other medical populations where modifiable variables are known to affect recovery outcomes. Such applications may be particularly relevant to medical conditions with known psychological and behavioral risk factors, such as cardiovascular disease (Flynn et al., 2019) and diabetes (Rode and Rode, 2018). This novel approach has the potential to significantly improve patient recovery outcomes across a range of medical conditions, through the process of interdisciplinary collaboration.

## Author Contributions

ED conducted the literature review and drafted the manuscript, with writing contributions from SJ. ED, SJ, NH, TF, MP, KR, FW, and MN reviewed and commented on the manuscript, contributing individual expert advice. ED, SJ, NH, TF, MP, KR, FW, and MN contributed to the conceptual development of the proposed multivariate tool. MN and FW conceived of and oversaw the project.

## Conflict of Interest

The authors declare that the research was conducted in the absence of any commercial or financial relationships that could be construed as a potential conflict of interest.
